# Supplementary material for: Down-Regulation of Rice Glutelin by CRISPR-Cas9 Gene Editing Decreases Carbohydrate Content and Grain Weight and Modulates Synthesis of Seed Storage Proteins during Seed Maturation
Source: Int J Mol Sci. 2023 Nov 29;24(23):16941. doi: 10.3390/ijms242316941 (PMC10707166; doi:10.3390/ijms242316941)
Supplement: Supplementary file 1 [file ijms-24-16941-s001.zip › Supplemetary Figures_Rev2023122.pptx]

## Slide 1
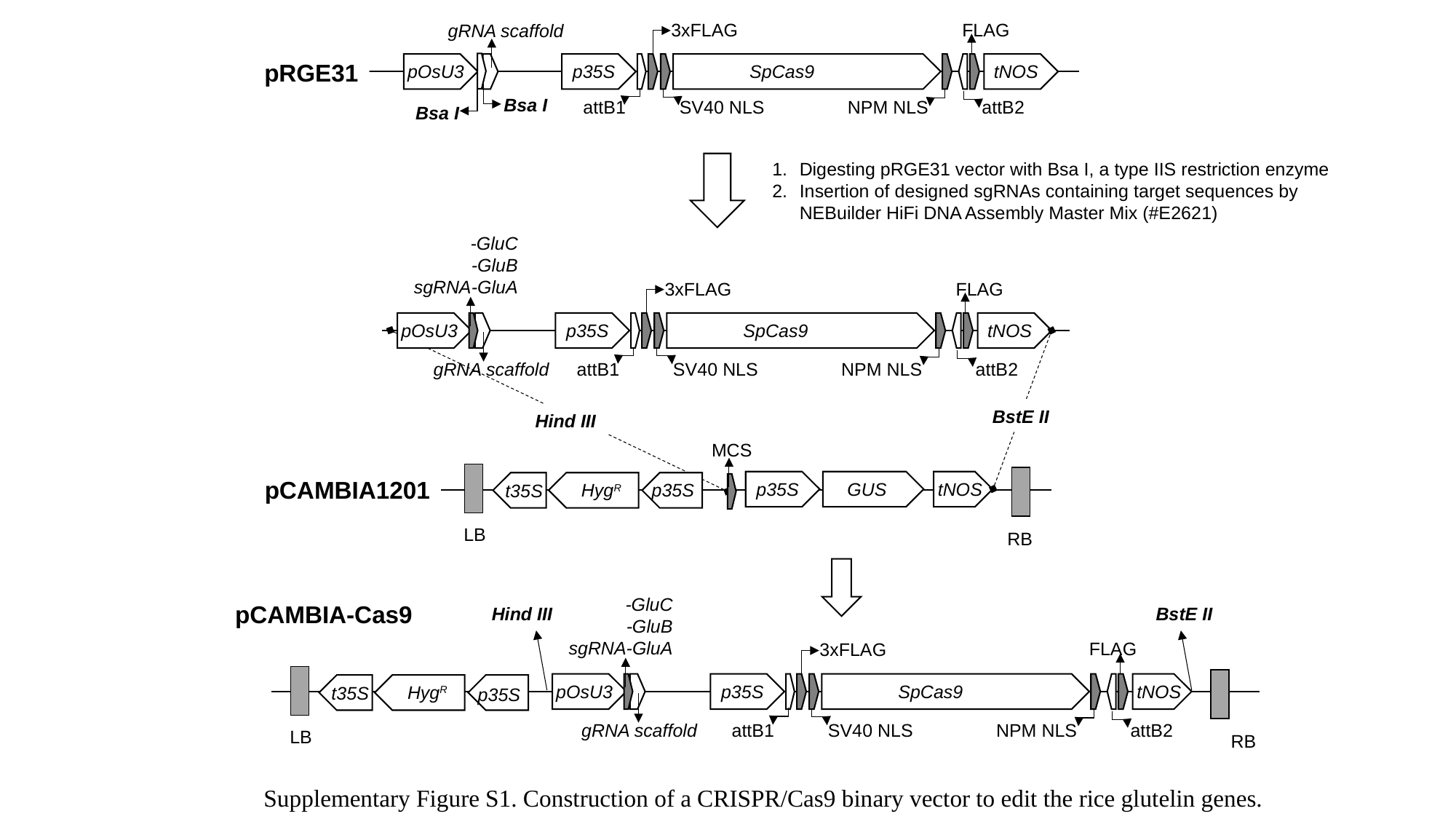

3xFLAG
FLAG
gRNA scaffold
pRGE31
pOsU3
p35S
SpCas9
tNOS
Bsa I
attB1
SV40 NLS
NPM NLS
attB2
Bsa I
Digesting pRGE31 vector with Bsa I, a type IIS restriction enzyme
Insertion of designed sgRNAs containing target sequences by NEBuilder HiFi DNA Assembly Master Mix (#E2621)
 -GluC
-GluB
sgRNA-GluA
3xFLAG
FLAG
pOsU3
p35S
SpCas9
tNOS
gRNA scaffold
attB1
SV40 NLS
NPM NLS
attB2
BstE II
Hind III
MCS
pCAMBIA1201
p35S
GUS
tNOS
HygR
p35S
t35S
LB
RB
 -GluC
-GluB
sgRNA-GluA
pCAMBIA-Cas9
Hind III
BstE II
FLAG
3xFLAG
pOsU3
p35S
SpCas9
tNOS
HygR
t35S
p35S
gRNA scaffold
attB1
SV40 NLS
NPM NLS
attB2
LB
RB
Supplementary Figure S1. Construction of a CRISPR/Cas9 binary vector to edit the rice glutelin genes.

## Slide 2
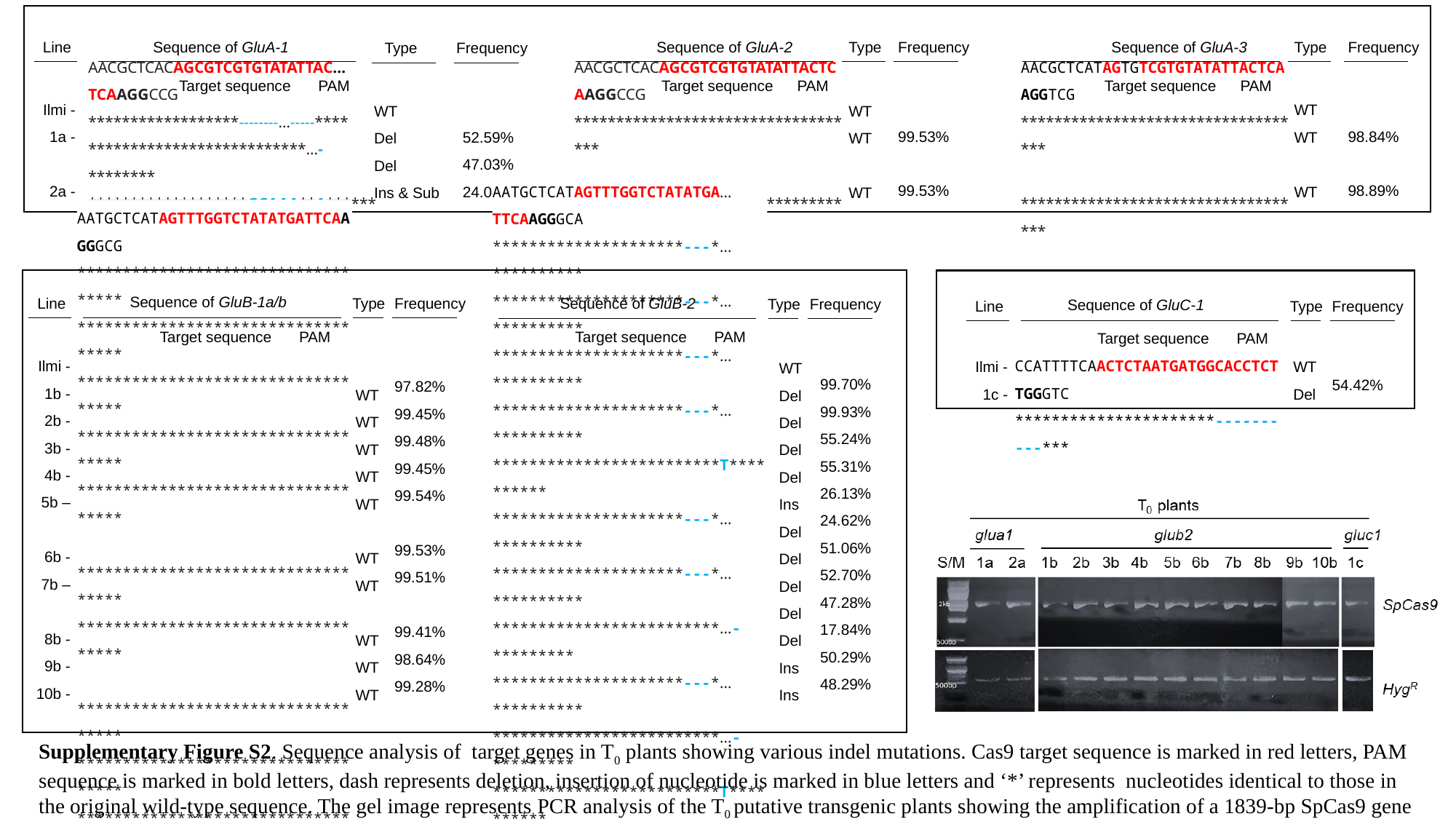

Line
Sequence of GluA-1
Sequence of GluA-2
Type
Frequency
Sequence of GluA-3
Type
Frequency
Type
Frequency
Target sequence
PAM
Target sequence
PAM
Target sequence
PAM
Ilmi -
1a -
2a -
WT
WT
WT
WT
Del
Del
Ins & Sub
WT
WT
WT
AACGCTCACAGCGTCGTGTATATTAC…TCAAGGCCG
******************--------…-----****
**************************…-********
*******************GCAAA**A*********
AACGCTCACAGCGTCGTGTATATTACTCAAGGCCG
***********************************
***********************************
AACGCTCATAGTGTCGTGTATATTACTCAAGGTCG
***********************************
***********************************
99.53%
99.53%
98.84%
98.89%
52.59%
47.03%
24.04%
Sequence of GluB-1a/b
Line
Type
Frequency
Sequence of GluB-2
Type
Frequency
Sequence of GluC-1
Line
Type
Frequency
Target sequence
PAM
Target sequence
PAM
Target sequence
PAM
CCATTTTCAACTCTAATGATGGCACCTCTTGGGTC
**********************----------***
Ilmi -
1b -
2b -
3b -
4b -
5b –
6b -
7b –
8b -
9b -
10b -
99.70%
99.93%
55.24%
55.31%
26.13%
24.62%
51.06%
52.70%
47.28%
17.84%
50.29%
48.29%
Ilmi -
1c -
WT
Del
54.42%
97.82%
99.45%
99.48%
99.45%
99.54%
99.53%
99.51%
99.41%
98.64%
99.28%
WT
Del
Del
Del
Del
Ins
Del
Del
Del
Del
Del
Ins
Ins
AATGCTCATAGTTTGGTCTATATGATTCAAGGGCG
***********************************
***********************************
***********************************
***********************************
***********************************
***********************************
***********************************
***********************************
***********************************
***********************************
AATGCTCATAGTTTGGTCTATATGA…TTCAAGGGCA
*********************---*…**********
*********************---*…**********
*********************---*…**********
*********************---*…**********
*************************T**********
*********************---*…**********
*********************---*…**********
*************************…-*********
*********************---*…**********
*************************…-*********
*************************T**********
*************************T**********
WT
WT
WT
WT
WT
WT
WT
WT
WT
WT
Supplementary Figure S2. Sequence analysis of target genes in T0 plants showing various indel mutations. Cas9 target sequence is marked in red letters, PAM sequence is marked in bold letters, dash represents deletion, insertion of nucleotide is marked in blue letters and ‘*’ represents nucleotides identical to those in the original wild-type sequence. The gel image represents PCR analysis of the T0 putative transgenic plants showing the amplification of a 1839-bp SpCas9 gene and 615-bp HygR gene. Bioneer 1kb DNA ladder (Cat.#D-1041) and 100 bp DNA ladder (Cat.#D-1030) were used as size marker.

## Slide 3
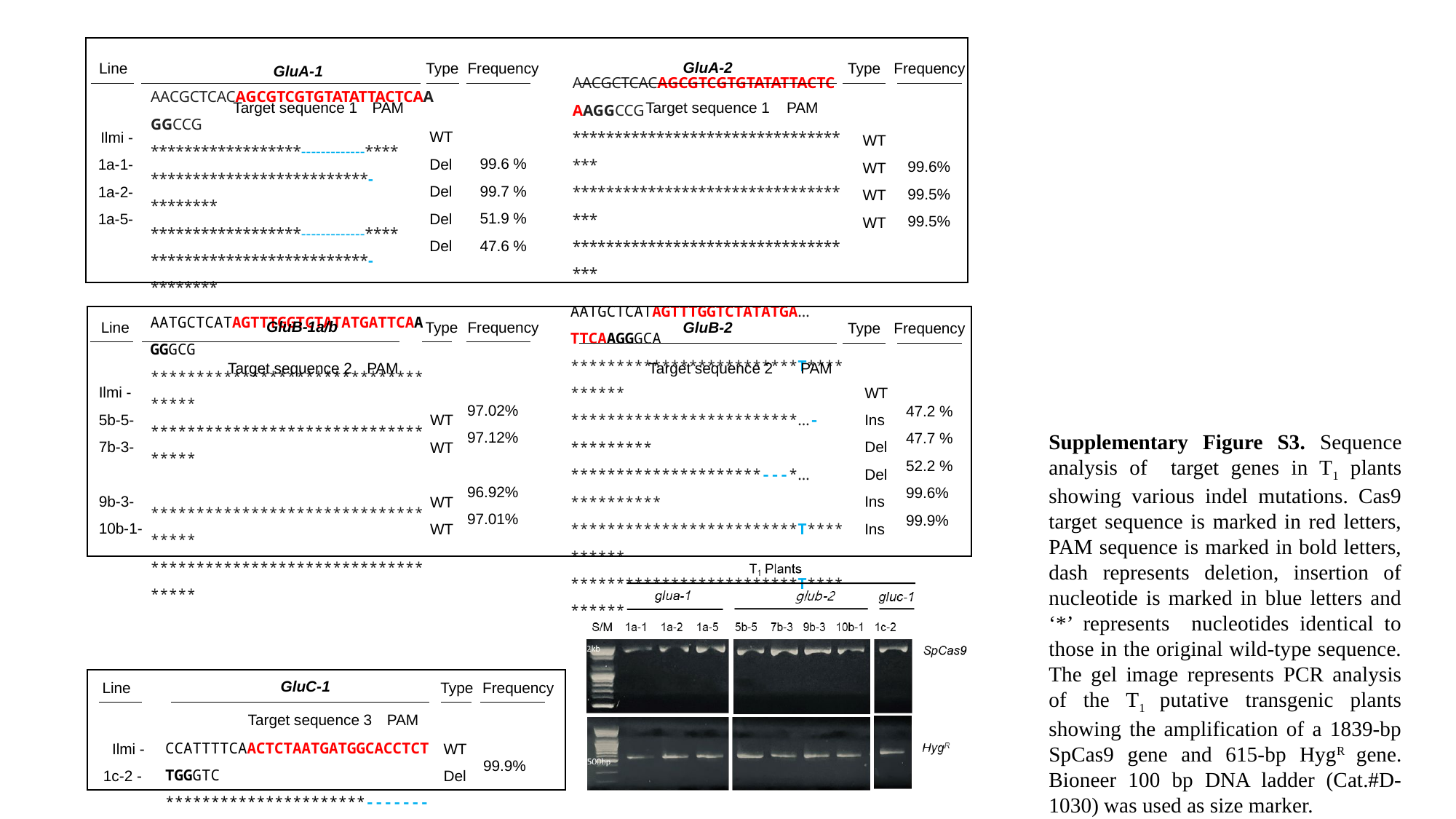

GluA-2
Line
Type
Frequency
Type
Frequency
GluA-1
Target sequence 1
PAM
Target sequence 1
PAM
WT
Del
Del
Del
Del
Ilmi -
1a-1-
1a-2-
1a-5-
WT
WT
WT
WT
AACGCTCACAGCGTCGTGTATATTACTCAAGGCCG
******************-------------****
**************************-********
******************-------------****
**************************-********
AACGCTCACAGCGTCGTGTATATTACTCAAGGCCG
***********************************
***********************************
***********************************
99.6 %
99.7 %
51.9 %
47.6 %
99.6%
99.5%
99.5%
GluB-1a/b
Line
Type
Frequency
GluB-2
Type
Frequency
47.2 %
47.7 %
52.2 %
99.6%
99.9%
Target sequence 2
PAM
Target sequence 2
PAM
97.02%
97.12%
96.92%
97.01%
Ilmi -
5b-5-
7b-3-
9b-3-
10b-1-
WT
Ins
Del
Del
Ins
Ins
AATGCTCATAGTTTGGTCTATATGATTCAAGGGCG
***********************************
***********************************
***********************************
***********************************
AATGCTCATAGTTTGGTCTATATGA…TTCAAGGGCA
*************************T**********
*************************…-*********
*********************---*…**********
*************************T**********
*************************T**********
WT
WT
WT
WT
Supplementary Figure S3. Sequence analysis of target genes in T1 plants showing various indel mutations. Cas9 target sequence is marked in red letters, PAM sequence is marked in bold letters, dash represents deletion, insertion of nucleotide is marked in blue letters and ‘*’ represents nucleotides identical to those in the original wild-type sequence. The gel image represents PCR analysis of the T1 putative transgenic plants showing the amplification of a 1839-bp SpCas9 gene and 615-bp HygR gene. Bioneer 100 bp DNA ladder (Cat.#D-1030) was used as size marker.
GluC-1
Line
Type
Frequency
Target sequence 3
PAM
CCATTTTCAACTCTAATGATGGCACCTCTTGGGTC
**********************----------***
99.9%
Ilmi -
1c-2 -
WT
Del

## Slide 4
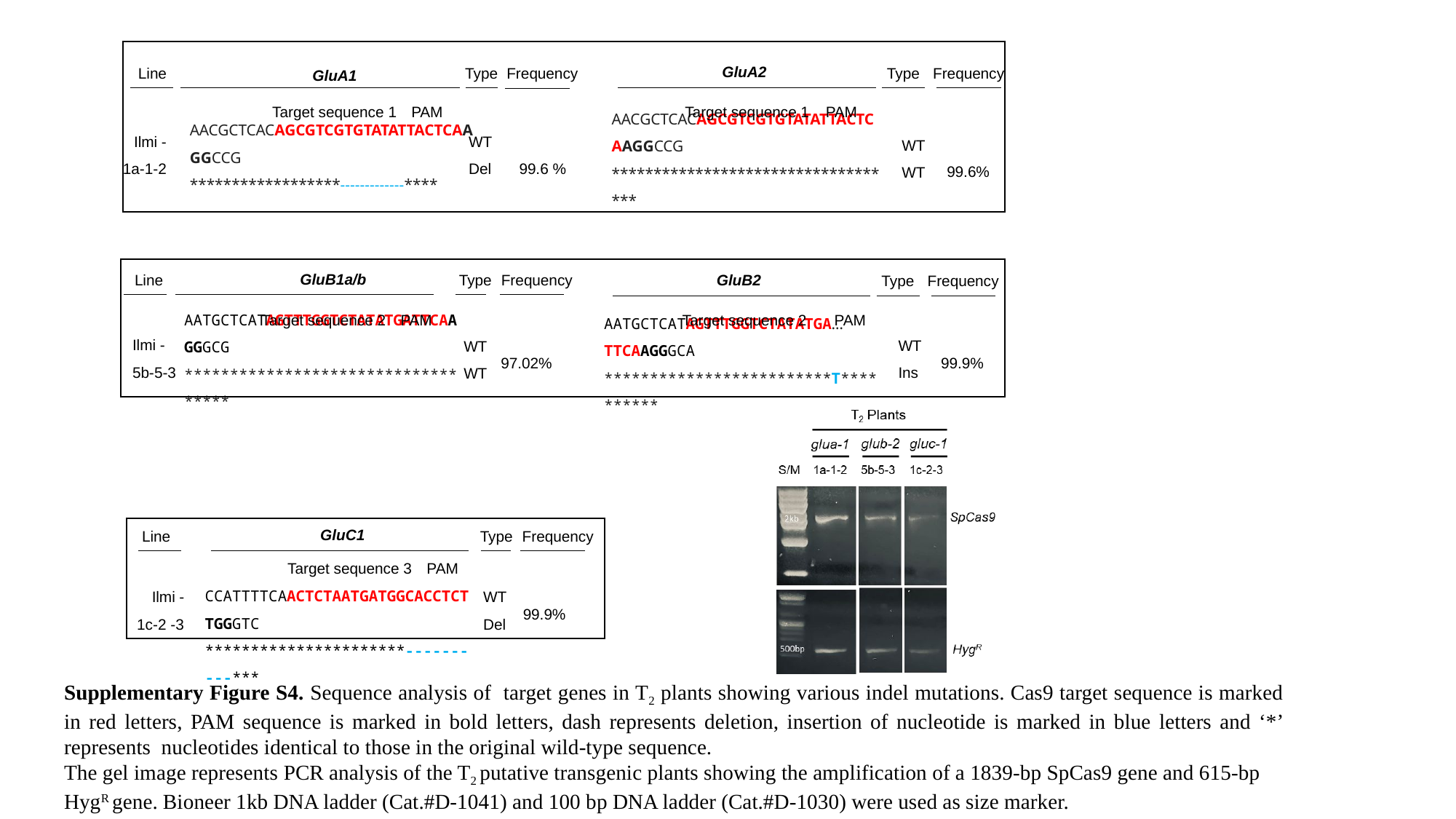

GluA2
Line
Type
Frequency
Type
Frequency
GluA1
Target sequence 1
PAM
Target sequence 1
PAM
WT
Del
Ilmi -
1a-1-2
WT
WT
AACGCTCACAGCGTCGTGTATATTACTCAAGGCCG
******************-------------****
AACGCTCACAGCGTCGTGTATATTACTCAAGGCCG
***********************************
99.6 %
99.6%
GluB1a/b
Line
Type
Frequency
GluB2
Type
Frequency
99.9%
Target sequence 2
PAM
Target sequence 2
PAM
97.02%
Ilmi -
5b-5-3
WT
Ins
WT
WT
AATGCTCATAGTTTGGTCTATATGATTCAAGGGCG
***********************************
AATGCTCATAGTTTGGTCTATATGA…TTCAAGGGCA
*************************T**********
GluC1
Line
Type
Frequency
Target sequence 3
PAM
CCATTTTCAACTCTAATGATGGCACCTCTTGGGTC
**********************----------***
99.9%
Ilmi -
1c-2 -3
WT
Del
Supplementary Figure S4. Sequence analysis of target genes in T2 plants showing various indel mutations. Cas9 target sequence is marked in red letters, PAM sequence is marked in bold letters, dash represents deletion, insertion of nucleotide is marked in blue letters and ‘*’ represents nucleotides identical to those in the original wild-type sequence.
The gel image represents PCR analysis of the T2 putative transgenic plants showing the amplification of a 1839-bp SpCas9 gene and 615-bp HygR gene. Bioneer 1kb DNA ladder (Cat.#D-1041) and 100 bp DNA ladder (Cat.#D-1030) were used as size marker.

## Slide 5
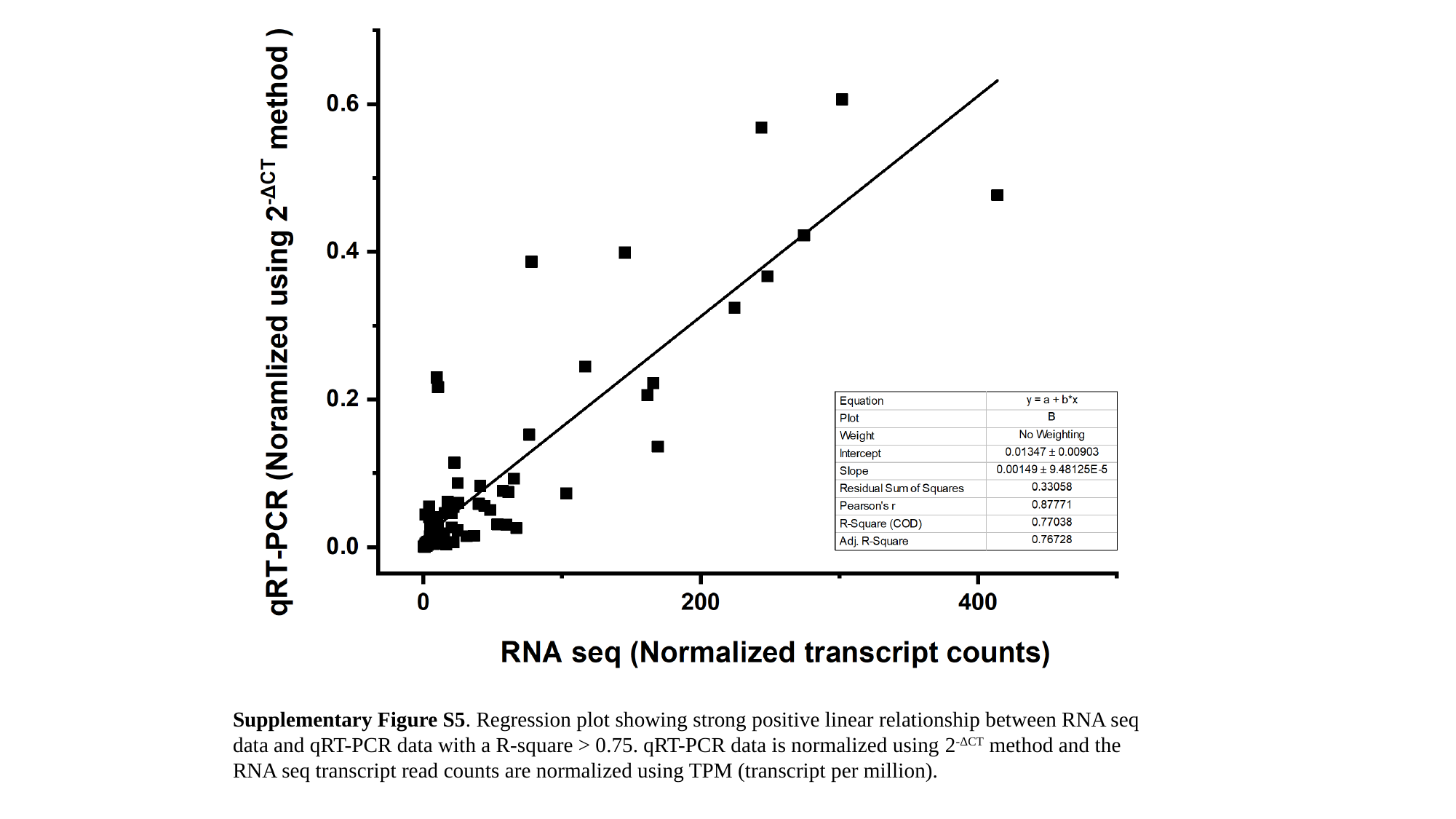

Supplementary Figure S5. Regression plot showing strong positive linear relationship between RNA seq data and qRT-PCR data with a R-square > 0.75. qRT-PCR data is normalized using 2-ΔCT method and the RNA seq transcript read counts are normalized using TPM (transcript per million).

## Slide 6
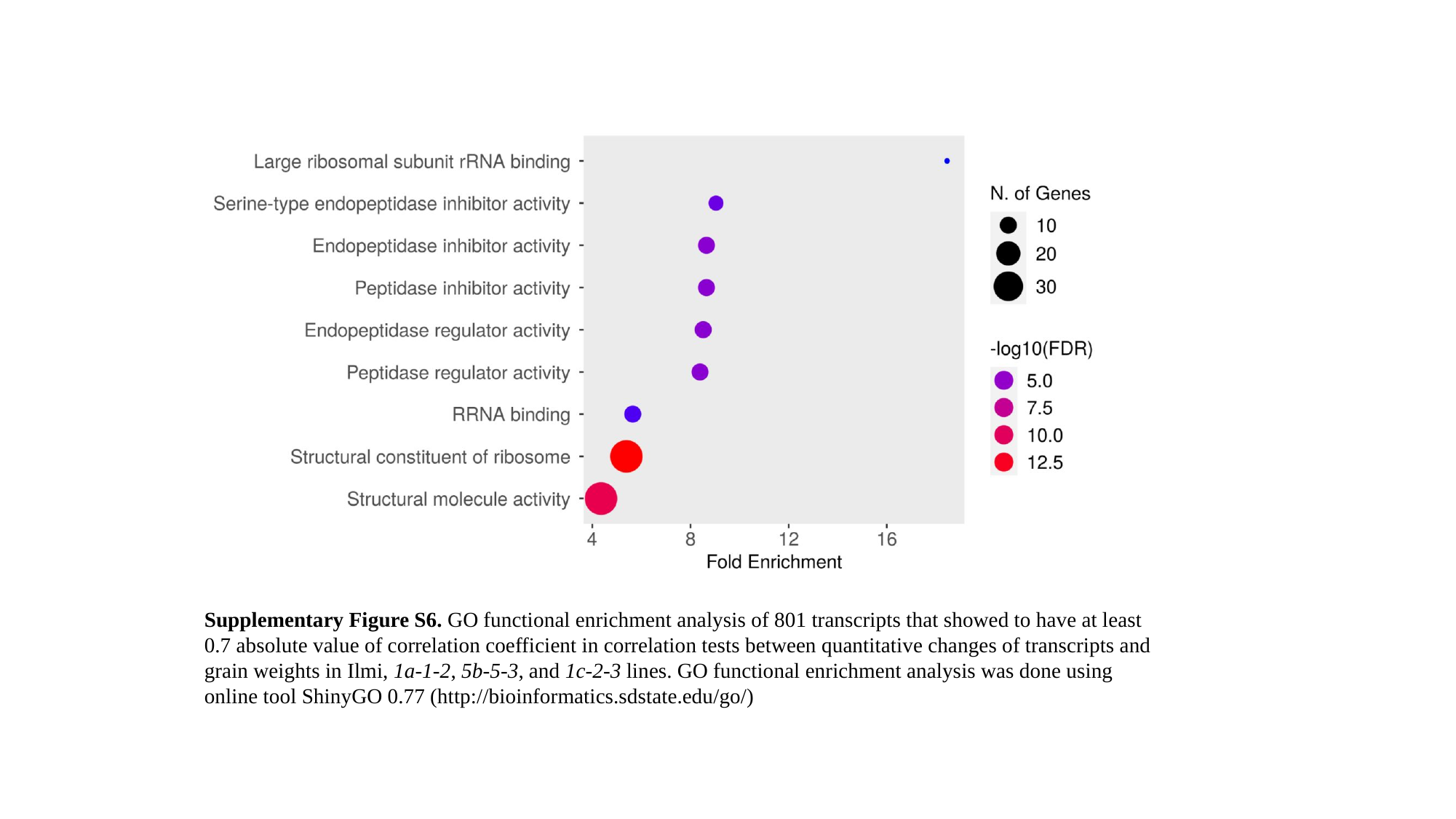

Supplementary Figure S6. GO functional enrichment analysis of 801 transcripts that showed to have at least 0.7 absolute value of correlation coefficient in correlation tests between quantitative changes of transcripts and grain weights in Ilmi, 1a-1-2, 5b-5-3, and 1c-2-3 lines. GO functional enrichment analysis was done using online tool ShinyGO 0.77 (http://bioinformatics.sdstate.edu/go/)

## Slide 7
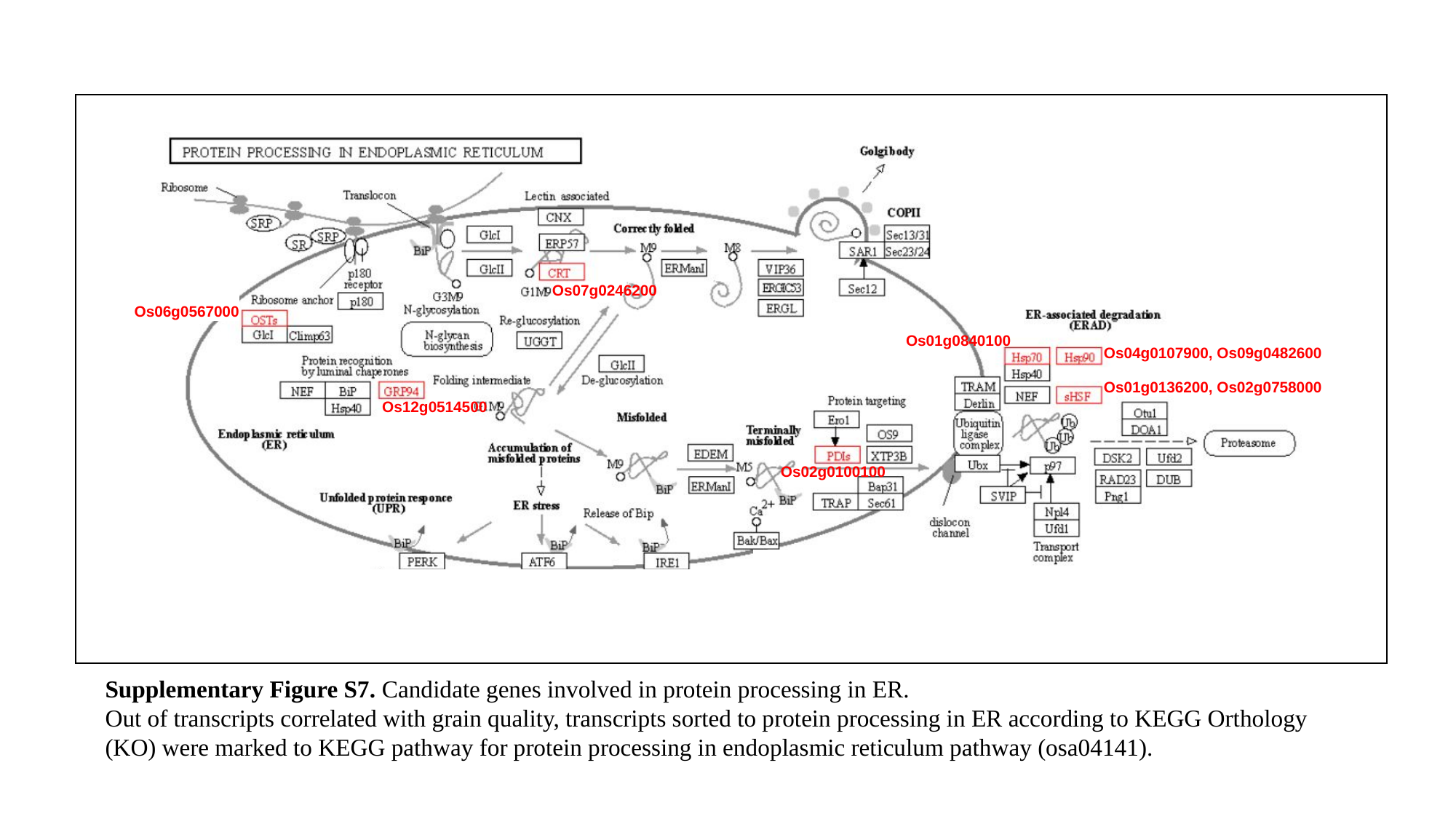

Os07g0246200
Os06g0567000
Os01g0840100
Os04g0107900, Os09g0482600
Os01g0136200, Os02g0758000
Os12g0514500
Os02g0100100
Supplementary Figure S7. Candidate genes involved in protein processing in ER.
Out of transcripts correlated with grain quality, transcripts sorted to protein processing in ER according to KEGG Orthology (KO) were marked to KEGG pathway for protein processing in endoplasmic reticulum pathway (osa04141).

## Slide 8
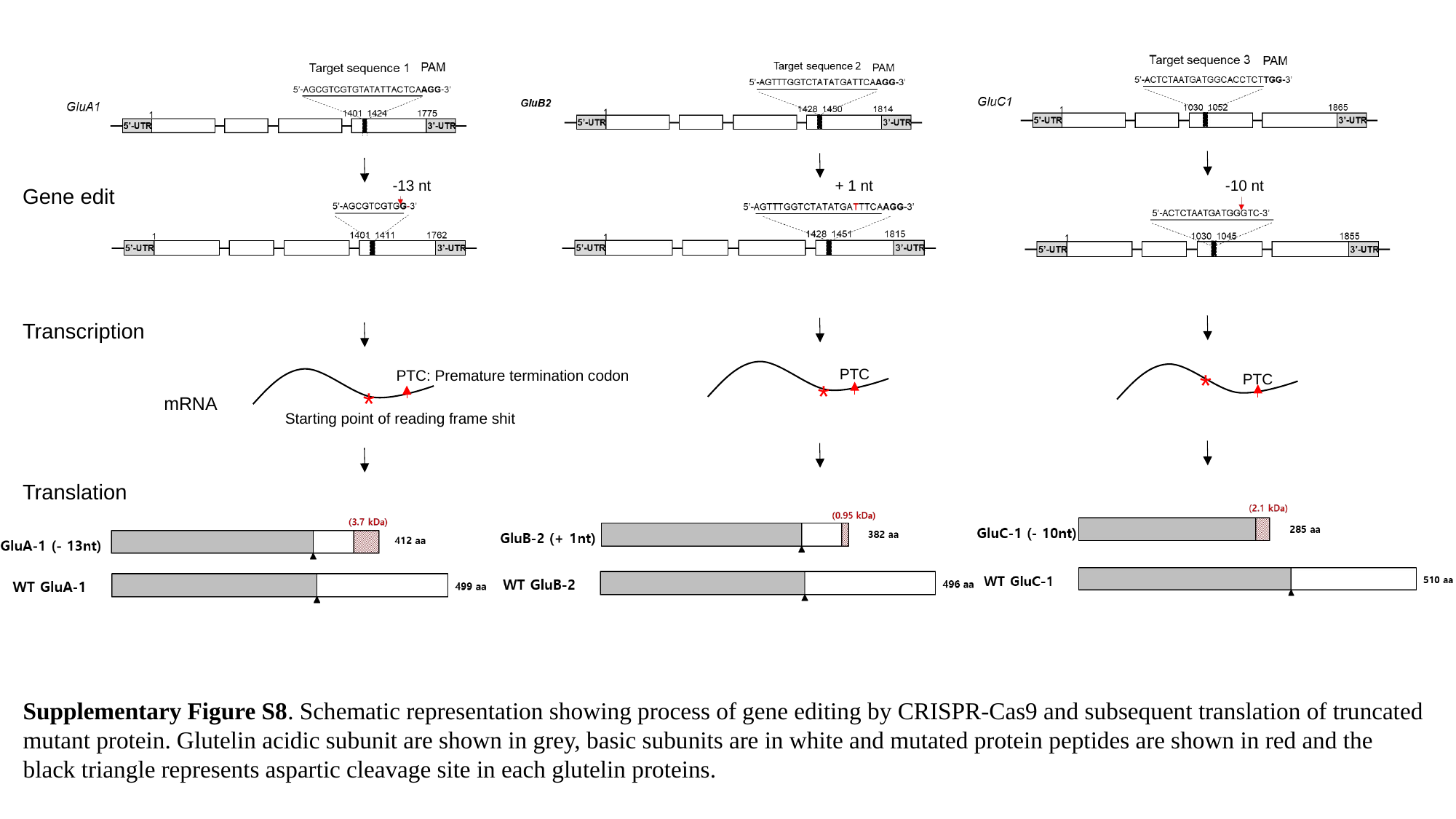

-13 nt
+ 1 nt
-10 nt
Gene edit
Transcription
PTC
PTC: Premature termination codon
*
PTC
*
*
mRNA
Starting point of reading frame shit
Translation
Supplementary Figure S8. Schematic representation showing process of gene editing by CRISPR-Cas9 and subsequent translation of truncated mutant protein. Glutelin acidic subunit are shown in grey, basic subunits are in white and mutated protein peptides are shown in red and the black triangle represents aspartic cleavage site in each glutelin proteins.
